# Nontuberculous Mycobacteria Infections and Anti–Tumor Necrosis Factor-α Therapy

**DOI:** 10.3201/eid1510.090310

**Published:** 2009-10

**Authors:** Kevin L. Winthrop, Eric Chang, Shellie Yamashita, Michael F. Iademarco, Philip A. LoBue

**Affiliations:** Oregon Health and Sciences University, Portland, Oregon, USA (K.L. Winthrop, E. Chang, S. Yamashita); US Public Health Service, Washington, DC, USA (M.F. Iademarco); Centers for Disease Control and Prevention, Atlanta, Georgia, USA (P.A. LoBue)

**Keywords:** Nontuberculous mycobacteria, tuberculosis and other mycobacteria, biologic therapy, tumor necrosis factor-α, adverse events, podcast, research

## Abstract

Patients receiving anti–tumor necrosis factor-α (anti–TNF-α) therapy are at increased risk for tuberculosis and other granulomatous diseases, but little is known about illness caused by nontuberculous mycobacteria (NTM) in this setting. We reviewed the US Food and Drug Administration MedWatch database for reports of NTM disease in patients receiving anti–TNF-α therapy. Of 239 reports collected, 105 (44%) met NTM disease criteria. Median age was 62 years; the majority of patients (66, 65%) were female, and most (73, 70%) had rheumatoid arthritis. NTM infections were associated with infliximab (n = 73), etanercept (n = 25), and adalimumab (n = 7); most patients were taking prednisone (n = 68, 65%) or methotrexate (n = 58, 55%) concurrently. *Mycobacteria avium* (n = 52, 50%) was most commonly implicated, and 9 patients (9%) had died at the time their infections were reported. A high rate of extrapulmonary manifestations (n = 46, 44%) was also reported.

## Abstract 2

Most infections were in rheumatoid arthritis patients, and 44% were extrapulmonary or disseminated.

Nontuberculous mycobacteria (NTM) are a large, diverse group of environmental organisms ubiquitous in water and soil (*1*). They cause a variety of diseases in humans, notably severe, protracted lung disease in patients with underlying lung disorders. Conditions such as bronchiectasis, emphysema, previous tuberculosis (TB) or other lung infections, cystic fibrosis, rheumatoid arthritis, and other chronic diseases with pulmonary manifestations can predispose a person to NTM pulmonary disease (*2*). In addition to lung infections, NTM cause skin and soft tissue infections, lymphadenitis (predominantly in young children), and disseminated disease in HIV-infected patients or others with severely compromised immune systems. The immunologic mechanism and related dysfunction that predispose persons to NTM disease are largely unknown, although defects in interleukin-12 or interferon-γ production are known to increase the risk for disseminated NTM disease in humans (*3*).

Although the epidemiology of NTM disease is not well described, the belief that these infections are increasing in prevalence, particularly among women, is widespread (*2*). Assessment of the epidemiology of these infections may be increasingly useful because newer forms of biologic, immunosuppressive therapies have become widely used for treating patients with rheumatoid arthritis, Crohn disease, and other autoimmune inflammatory conditions. Many of these conditions are associated with lung manifestations known to be associated with NTM pulmonary infections (*2*).

To date, TB and NTM infections and concurrent biologic therapies that inhibit tumor necrosis factor-α (TNF-α) have been reported. These therapies include infliximab (Remicade; Centocor, Malvern, PA, USA), etanercept (Enbrel; Immunex, Seattle, WA, USA), and adalimumab (Humira; Abbott Biotechnology, Abbott Park, IL, USA), which have been approved in the United States and elsewhere to treat patients with rheumatoid arthritis and selected other autoimmune inflammatory diseases (*4*). Because TNF-α is integral to granuloma generation and maintenance (*5*,*6*), patients using these agents are at increased risk for granulomatous infections, including activation of latent TB infection (*7*,*8*).

The US Food and Drug Administration (FDA) postmarketing surveillance system (MedWatch) (www.fda.gov/medwatch) collects voluntary reports of adverse drug events from physicians. The most recent review of this system in 2004 for reports of granulomatous infections that occurred during TNF-α blockade found that mycobacteria disease was more common than other granulomatous diseases; TB was reported 5–10× more frequently than NTM, dimorphic fungi, and other intracellular infections in this setting (*7*). (Although this program does not specifically target participation outside the United States, it also includes nondomestic case reports.) Subsequently, much attention has been focused on prevention of TB in patients who are using anti–TNF-α agents. To date, little is known regarding the types and relative frequencies of NTM infections that occur in such patients.

We recently conducted a survey among infectious disease physicians within the Emerging Infections Network of the Infectious Diseases Society of America (IDSA). This survey suggested that cases of NTM disease associated with anti–TNF-α therapy occur twice as frequently as cases of TB associated with anti–TNF-α therapy in the United States (*9*). NTM infections are likely underreported to the FDA, relative to TB, for a variety of reasons (*10*). NTM disease is generally insidious, sometimes difficult to diagnose, and is not reportable to health authorities. Accordingly, we reviewed the MedWatch database for NTM reports through January 1, 2007, to evaluate whether these case reports met clinical case criteria, to describe their clinical spectrum and outcome, and to evaluate the relative reporting frequency of cases among the different anti–TNF-α agents now in widespread use.

## Methods

At our request, FDA searched its MedWatch database for NTM cases reported among patients using adalimumab, infliximab, or etanercept through January 1, 2007. Using the search terms nontuberculous mycobacteria infections, atypical mycobacteria infections, and leprous infections, FDA compiled all domestic and foreign reports with keywords matching at least 1 of these search terms. The reports were redacted to remove any identifying information and sent to us for review. Because we sought to review only cases involving environmental NTM, cases caused by *Mycobacterium leprae* (leprosy) were excluded (n = 5). We reviewed all reports and extracted the following data: etiologic organism, anti–TNF-α drug, and concomitant immunosuppressive drugs used at the time of infection diagnosis, clinical and radiographic data, death or hospitalization during infection treatment, and time between beginning drug treatment and infection diagnosis.

To define pulmonary disease, we used the American Thoracic Society (ATS)/IDSA case definition in which patients must have >2 sputum samples with NTM (or a single isolate in the case of bronchoscopy specimens) coexistent with appropriate radiographic findings and symptoms (*2*). If cases met these criteria they were deemed confirmed. If not enough information was provided for the case definition to be considered, they were deemed probable or unknown based on the consensus opinion of 3 physicians from the Division of Infectious Diseases at Oregon Health and Sciences University. For extrapulmonary disease, patients with NTM isolated from normally sterile sites were considered to have confirmed cases. Disease reports that included infection with *M*. *tuberculosis* or organisms other than mycobacteria were excluded.

### Data Analysis

All data were entered into Epi Info version 3.4.3 (Centers for Disease Control and Prevention, Atlanta, GA, USA). Two-by-two comparisons among variables were made by using Mantel-Haenszel odds ratios (ORs) and Fisher exact test p values. We did not attempt to calculate or compare NTM incidence rates among different anti–TNF-α products because the MedWatch database does not include drug exposure denominator data.

## Results

**Figure 1:**
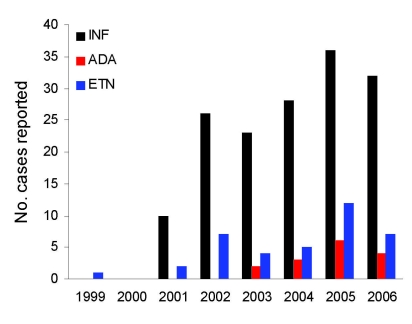
Case reports of nontuberculous mycobacteria in patients using antitumor necrosis factor-α (TNF-α) therapy, US Food and Drug Administration MedWatch database, 1999–2006. Cases are reported by each full year of data reporting for each anti-TNF agent. Reported cases for all agents were most numerous in 2005. INF, infliximab; ADA, adalimumab; ETN, etanercept.

There were 239 reports of NTM infection in patients who were receiving anti–TNF-α therapy. Most reports were for patients receiving infliximab (n = 174, 75%), followed by etanercept (n = 41, 17%), and adalimumab (n = 19, 8%). One case was reported in 1999 (patient used etanercept); numbers of reported infections among those using each product increased in 2001 and thereafter. Reported cases among those using each of the 3 drugs were highest in 2005 (Figure 1). Of these reports, only 76 (32%) met either ATS/IDSA pulmonary disease criteria or our case definition for extrapulmonary disease. An additional 29 (12%) cases were judged to be probable cases, but the reports did not contain enough clinical or radiographic information to determine whether patients met ATS/IDSA NTM disease criteria. In other instances, the reports were either clearly not of cases of NTM disease (n = 27, 11%) or could not be determined (n = 95, 40%) because of a lack of microbiologic data, unclear reporting, or duplicate reports (n = 12, 5%). Of the 244 reports, 76 (31%) were from outside the United States (Europe, n = 40; Japan, n = 21; Canada, n = 4; Israel, n = 1; South Africa, n = 1; not specified, n = 9). Of patients with confirmed and probable cases (n = 105), a similar proportion (n = 35, 33%) were from outside the United States; most of these were from Europe (n = 15) or Japan (n = 12).

**Figure 2:**
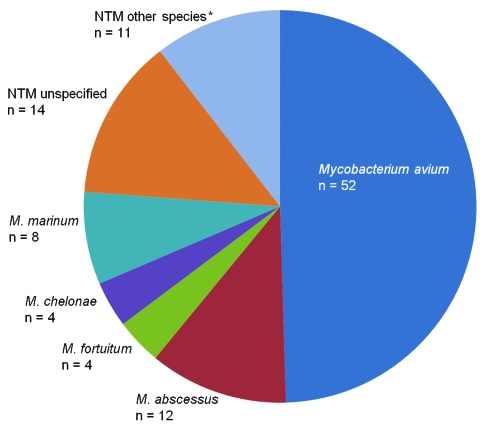
Reported causes of 105 confirmed and probable nontuberculous mycobacteria (NTM) infections associated with antitumor necrosis factor-α agents, US Food and Drug Administration MedWatch database, 1999–2006. *Other species include *Mycobacterium* *kansasii* (n = 3), *M*. *xenopi* (n = 3), *M*. *haemophilum* (n = 2), and *M*. *mucogenicum* (n = 1).

Of the 105 confirmed or probable cases, most were in women (n = 66, 65%), and the median age was 63 years (range 20–90 years). The anti–TNF-α agents reported for these patients included infliximab (n = 73, 69%), etanercept (n = 25, 24%), and adalimumab (n = 7, 7%). *M*. *avium* was the most common etiologic organism reported (n = 52, 49%), followed by rapidly growing mycobacteria (n = 20, 19%), and *M*. *marinum* (n = 8, 8%) (Figure 2). Nine patients (9%) had died by the time their case was reported, and 64 (61%) had NTM adverse events that resulted in hospitalization. The most common underlying medical indication for anti–TNF-α therapy was rheumatoid arthritis (n = 73, 75%), followed by other inflammatory diseases (Table 1). Sixty-eight (65%) patients received concomitant prednisone, and 58 (55%) received methotrexate at the time of their report. Twenty-five (24%) patients reportedly had >1 of the following conditions: bronchiectasis (n = 5, 5%), chronic obstructive pulmonary disease (n = 11, 10%), diabetes mellitus (n = 5, 5%), and rheumatoid lung (n = 4, 4%). Median time between anti–TNF-α agent start date and infection diagnosis was available for only 68 (65%) of the patients. For adalimumab (n = 5), the interval was 18 weeks (range 4–94 weeks), for etanercept (n = 22) it was 35 weeks (range 0–288 weeks), and for infliximab (n = 41) it was 43 weeks (range 2–200 weeks).

**Table 1:** Reported diseases associated with anti–TNF-α therapy and therapy implicated for 105 cases of NTM disease, US Food and Drug Administration MedWatch database, 1999–2006*

Disease	Infliximab	Etanercept	Adalimumab
Rheumatoid arthritis (n = 73)	51	17	5
Ankylosing spondylitis (n = 5)	1	3	1
Psoriasis (n = 4)	2	2	0
Crohn disease (n = 8)	8	0	0
Other (n = 15)†	11	3	1

*TNF-α, tumor necrosis factor-α; NTM, nontuberculous mycobacteria. †Includes Wegener granulomatosis (n = 2), dermatomyositis (n = 1), uveitis (n = 1), juvenile rheumatoid arthritis (n = 1), and not reported (n = 10).

The pulmonary region (n = 59, 56%) was the most frequently reported site of disease; the remainder of infections were extrapulmonary or disseminated (Table 2). Compared with patients with extrapulmonary NTM disease, patients with pulmonary NTM disease were more likely to have underlying rheumatoid arthritis (OR 3.6, 95% confidence interval [CI] 1.5–8.8, p<0.01) and more likely to be infected with *M*. *avium* (OR 11.0, 95% CI 4.4–27.9, p<0.01). Reported cases of pulmonary NTM disease were also more likely to be in female patients (OR 2.3, 95% CI 1.0–5.3, p = 0.04) (Table 3). After we adjusted for differences in sex in a stratified analysis, rheumatoid arthritis remained associated with pulmonary disease (adjusted OR 3.4, 95% CI 1.4–8.3, p = 0.01). There were no significant differences between the proportions of pulmonary disease, disseminated disease, death, or etiologic organisms reported for patients who used infliximab and those who used etanercept. Overall, infliximab users were more likely to be using methotrexate (OR 3.0, 95% CI 1.2–8.0, p = 0.02) than were etanercept users (63% vs. 36%). Prednisone use did not differ between the 2 groups; 50 (68%) of infliximab-treated patients and 14 (56%) of etanercept-treated patients were using prednisone at the time of their report (p = 0.19).

**Table 2:** Sites of infection for 105 reported anti–TNF-α therapy–associated cases of NTM disease, US Food and Drug Administration MedWatch database, 1999–2006*

Site	No. (%) cases
Pulmonary region	59 (56)
Skin or soft tissue	27 (26)
Bone or joint	10 (9)
Disseminated	8 (8)
Eye	1 (1)

*TNF-α, tumor necrosis factor-α; NTM, nontuberculous mycobacteria.

**Table 3:** Characteristics of 105 pulmonary and nonpulmonary anti–TNF-α therapy–associated cases of NTM disease, US Food and Drug Administration MedWatch database, 1999–2006*

Characteristic	Pulmonary (n = 59), no. (%)	Extrapulmonary (n = 46), no. (%)
*Mycobacterium avium*	43 (73)	9 (20)†
RGM	6 (10)	15 (33)†
Age, y	61	63
Female patient	41 (73)	25 (54)†
Rheumatoid arthritis	48 (81)	25 (54)†
Infliximab	40 (68)	33 (72)
Etanercept	13 (22)	12 (26)

*TNF-α, tumor necrosis factor-α; NTM, nontuberculous mycobacteria; RGM, rapidly growing mycobacteria. †p<0.05 for comparison of pulmonary disease and extrapulmonary disease.

For reported NTM adverse events that did not meet either ATS/IDSA case criteria or our probable case designation, similar analyses were performed. These persons were similar to persons with cases that met confirmed or probable case definitions. They did not differ with regard to sex, underlying medical condition, anti–TNF-α drugs used, mortality rate, or the ratio of pulmonary to extrapulmonary disease manifestations. However, these patients were less likely to be using concomitant prednisone (65% for patients with cases vs. 43% for patients without cases; OR 2.5, 95% CI 1.4–4.2, p<0.01) or concomitant methotrexate (55% for patients with cases vs. 37% for patients without cases; OR 2.1, 95% CI 1.3–3.6, p<0.01).

## Discussion

We reviewed the FDA MedWatch database for reports of NTM infections in patients using anti–TNF-α therapies. We found several hundred reported events; most had occurred in the 5 years since the previous review of this database (*7*). Our study scrutinized clinical features of NTM infections reported in the database and characterized the microbiologic and clinical features of NTM disease in a substantial number of patients who were receiving anti–TNF-α therapy. Similar to patients with anti–TNF-α–associated TB, patients frequently had extrapulmonary disease and most reported use of infliximab. *M*. *avium* was the most common etiologic agent reported; it was associated with pulmonary disease in elderly, female patients with rheumatoid arthritis.

To date, much of the available information regarding opportunistic infectious complications of anti–TNF-α therapy in the United States has been derived from the passive MedWatch surveillance system, a voluntary system subject to underreporting. The number of NTM reports associated with anti–TNF-α therapy has increased substantially in this database since publication of the initial MedWatch analysis by Wallis et al (*7*), in which the authors reviewed cases reported through September 2002 and before FDA approval of adalimumab (etanercept and infliximab gained FDA approval in the fall of 1998, and adalimumab gained approval at the end of 2002). Unlike that review, we examined clinical details of the cases in these reports to ascertain whether such patients actually met disease criteria. Because the respiratory tract is a nonsterile body site, pulmonary specimens can yield environmental organisms such as NTM in healthy persons. Accordingly, ATS and IDSA collaboratively published clinical criteria that must be met to determine whether a patient with pulmonary NTM isolates actually has NTM disease (*2*). Although we were able to apply this definition in some instances, our experience highlighted one of the limitations of the FDA MedWatch database: frequently a paucity of pertinent clinical details were reported. Accordingly, we found a high percentage of cases in MedWatch reports that did not meet disease criteria either because information was not reported or because some patients simply did not have reported pathologic conditions or other clinical criteria consistent with disease.

In our review, most patients with anti–TNF-α–related NTM were elderly women with rheumatoid arthritis. This finding is probably explained by several factors. First, rheumatoid arthritis is the most prevalent autoimmune inflammatory diseases for which anti–TNF-α therapies are approved (*4*). Approximately 0.5%–1.0% of the US population has rheumatoid arthritis (*11*), and >40% of rheumatoid arthritis patients have been treated with these therapies (*12*). Second, rheumatoid lung disease, which can include bronchiolitis and bronchiectasis, develops in ≈10% of these patients. These and other lung disorders are known to increase the risk for NTM disease. Third, the age and sex distribution of patients in this report mirrors that for patients with pulmonary NTM disease and rheumatoid arthritis independent of anti–TNF-α therapy. NTM disease and rheumatoid arthritis are more common in women >50 years of age (*2*,*11*). Fourth, a high percentage of patients who receive anti–TNF-α therapy are known to have serious medical conditions, some of which might increase the risk for NTM disease (*13*). In our series, >15% of patients were reported to have chronic obstructive pulmonary disease or bronchiectasis, which are known risk factors for pulmonary NTM disease.

Nearly half the patients in our series had extrapulmonary disease. In patients with TB, TNF inhibition is known to increase the risk for extrapulmonary and disseminated disease manifestations (*14*,*15*). Similar proportions of such disease were reported in our series of NTM patients. Similar to reports of TB, reports of NTM disease in the database were more numerous for persons who used infliximab. We were not able to access treatment start data for each of these agents because such information is proprietary, although etanercept and infliximab have been used more extensively than the more recently approved adalimumab (*4*).

Although no study has directly compared the risk for NTM disease between users of infliximab and users of other anti–TNF-α agents, use of infliximab may pose greater risk for NTM disease. If true, the risk could be caused by the drug itself or differences in the characteristics of patients given infliximab relative to users of the other anti–TNF-α compounds (*4**,**7*,*8*). For example, in our current series, infliximab users were more likely to be concomitantly using methotrexate at the time of diagnosis. The FDA database is limited because physician case reports are voluntary and no denominator data with regard to drug exposure are collected. For these reasons, we did not attempt to calculate or compare rates of NTM disease among various anti–TNF-α agents or among reporting years of this study. Thus, firm conclusions cannot be made regarding the comparative risk for use of these agents.

The US Centers for Disease Control and Prevention, the British Thoracic Society, and others have published recommendations describing the role of latent TB screening and treatment before use of anti–TNF-α therapy (*16*–*19*). Although screening can decrease the risk for TB in such patients (*20*), with the incumbent risk of illness and disease transmission, it is less clear what should be done to prevent NTM disease occurrence or progression in patients who use these compounds. Given the long median periods between the start of drug use and disease diagnosis within this case series, for many of these patients, NTM disease likely had been newly acquired during anti–TNF-α therapy.

Alternately, given the slow progression and insidious nature of pulmonary NTM disease, some of the patients in this series likely had existing but undiagnosed pulmonary NTM disease before starting their anti–TNF-α therapy. This likelihood raises the question whether patients should be screened for NTM disease before initiating anti–TNF-α therapy. According to published guidelines, all patients in the United States should be screened for latent TB infection before receiving therapy, a screening that includes a chest radiograph. Although abnormalities on such a radiograph could trigger sputum evaluation for TB and NTM, a chest radiograph is not sufficiently sensitive for detecting bronchiectasis or other lung abnormalities associated with NTM disease. If not previously obtained, clinicians could consider obtaining a noncontrast chest computed tomography scan before administering therapy to any patient with a history of bronchiectasis or other architectural lung disease, chronic unexplained cough, or abnormalities noted on their screening chest radiograph suggestive of NTM disease (e.g., reticulonodular infiltrate). If chest computed tomography suggests possible NTM disease, further pulmonary testing with sputum or other samples obtained by bronchoscopy would be indicated to rule out active NTM disease before initiating anti–TNF-α therapy (*21*).

Because most physicians would be reluctant to prescribe anti–TNF-α therapy to patients with known or obvious active infections, we suspect that most extrapulmonary infections (primarily soft tissue infections) in our series likely developed in patients after they began anti–TNF-α therapy. It is not clear what risk factors predisposed patients to these infections in our series and unlikely that such complications during therapy can be screened for or prevented.

There are increasing numbers of case reports of pulmonary NTM disease for patients using anti–TNF-α therapy (*9*,*22*,*23*). In some of these patients, pulmonary NTM disease progressed while they were receiving anti–TNF-α therapy, despite aggressive antimycobacterial treatment. Most patients in our series were hospitalized for their infections, and although <10% died by the time their case was reported, we suspect that follow-up beyond the time of the event report would indicate that a greater number of deaths occurred during therapy. Given the serious illnesses and deaths caused by these infections, whether anti–TNF-α therapy can be safely continued during antimycobacterial therapy is not clear. It is also not evident when it would be safe to reinstitute anti–TNF-α therapy in such patients.

We believe that our review of the FDA MedWatch database suggests that NTM represents a serious and severe granulomatous complication that can occur during anti–TNF-α therapy. However, lack of denominators for persons treated with these drugs precludes calculation of rates, and without a control group, it is not possible to definitively conclude that anti–TNF-α therapy causes or is associated with NTM disease. This finding is further complicated by potential confounders such as other immunosuppressive therapy and predisposing conditions among the population being studied. For example, epidemiologic features of patients who use anti–TNF-α drugs are similar to those who are at risk for NTM pulmonary disease in the absence of these drugs (i.e., elderly women, many of whom who have underlying lung disease). Nevertheless, our findings are useful because to date, the possibility of NTM disease has been underreported for patients who use anti–TNF-α therapies. Our findings highlight that these cases are occurring in such patients, often with devastating outcomes. Future population-based studies are necessary to determine risks for such complications and to define preventive and therapeutic strategies for such patients. For now, clinicians should remain vigilant for these and other types of serious infections that occur in patients using these compounds.
